# Zinc in Multiple Sclerosis: A Systematic Review and Meta-Analysis

**DOI:** 10.1177/1759091416651511

**Published:** 2016-06-09

**Authors:** Mikkel Bredholt, Jette Lautrup Frederiksen

**Affiliations:** 1 Department of Neurology, Rigshospitalet – Glostrup, University of Copenhagen, Glostrup, Denmark

**Keywords:** multiple sclerosis, zinc, meta-analysis, serum, plasma, matrix metalloproteinases

## Abstract

In the last 35 years, zinc (Zn) has been examined for its potential role in the disease multiple sclerosis (MS). This review gives an overview of the possible role of Zn in the pathogenesis of MS as well as a meta-analysis of studies having measured Zn in serum or plasma in patients with MS. Searching the databases PubMed and EMBASE as well as going through reference lists in included articles 24 studies were found measuring Zn in patients with MS. Of these, 13 met inclusion criteria and were included in the meta-analysis. The result of the meta-analysis shows a reduction in serum or plasma Zn levels in patients with MS with a 95% CI of [−3.66, −0.93] and a *p* value of .001 for the difference in Zn concentration in μM. One of six studies measuring cerebrospinal fluid, Zn levels found a significant increase in patients with MS with controls. The studies measuring whole blood and erythrocyte Zn levels found up to several times higher levels of Zn in patients with MS compared with healthy controls with decreasing levels during attacks in relapsing-remitting MS patients. Future studies measuring serum or plasma Zn are encouraged to analyze their data through homogenous MS patient subgroups on especially age, sex, and disease subtype since the difference in serum or plasma Zn in these subgroups have been found to be significantly different. It is hypothesized that local alterations of Zn may be actively involved in the pathogenesis of MS.

## Introduction

Multiple sclerosis (MS) is categorized as an autoimmune disease and is potentially one of the most common causes of neurological disability in young adults. The disease can generally be divided into three clinical subtypes: relapsing-remitting MS (RRMS), secondary progressive MS (SPMS), and primary progressive MS (PPMS), although studies question the homogeneity of the disease with especially PPMS having a different clinical course and a seemingly different pathogenesis (Foong et al., 2000; Lassmann et al., 2001). Formation of the sclerotic plaques of which the disease gets its name represents the end stage of a process involving inflammation, demyelination and remyelination, oligodendrocyt depletion, and astrogliosis as well as neuronal and axonal degeneration. The inflammation may impede the proper propagation in three ways: inflammatory mediators directly impede conduction in intact axons, the demyelination of axons by microglia thereby compromising the ability of proper conduction of the axon, or axonal transection (Compston and Coles, 2008). The etiology of the disease is not known though it is clear that it is caused by a complex interplay between genes and environment (Sadovnick and Ebers, 1993). The strongest genetic associations have been found to be in the major histocompatibility complex with HLA-DRB1 to have the strongest effect (Patsopoulos et al., 2013). Other genes have been identified as well, with Zn-ion binding genes involved in the expression of IFN-γ being able to predict the clinical outcome in RRMS patients with 88.9% accuracy (Achiron et al., 2007).

### Zinc

Zn is an essential trace element for all organisms, and Zn level alterations have consequences on particularly the nervous, the reproductive and the immune system. Being a cofactor of more than 300 enzymes including matrix metalloproteinases (MMPs) and a component of an even greater amount of proteins including Myelin Basic Protein, Zn emphasizes its important role in human health (Vallee and Falchuk, 1993; Berlet et al., 1994). Zn's plasma concentration is 12 to 16 µM and is nearly completely protein-bound with 80% bound to albumin and 20% bound to α2-macroglobulin (Scott and Bradwell, 1983). Zn is an abundant metal in the brain and is mainly found in presynaptic vesicles in neurons. With Zn deficiency being able to induce apoptosis in neural cells as well as high levels of Zn being neurotoxic, it is clear that efficient Zn homeostasis in the brain is important (Yokoyama et al., 1986; Adamo et al., 2010).

#### Zn and the immune system

Zn deficiency decreases several functions in the immune system including peripheral T-cell count, T-helper cell function, cytotoxic T-cell activity, NK-cell activity, and macrophage and neutrophil functions (Rink and Gabriel, 2000). Even though these effects seem contradictory of Zn deficiency being involved in the exacerbation of MS as an autoimmune disease, a study by Kitamura et al. (2006) showed that the intracellular reduction of Zn is an activator of dendritic cell maturation resulting in increased cell-surface expression of MHC and costimulatory molecules, potentially resulting in unwanted activation of T-cells and T-cell mediated autoimmunity. Elevation of Zn alone can induce the expression of the high-affinity receptor for IL-2, important for the proliferation and differentiation of CD4 + and CD8 + lymphocytes to effector-cells, as well as being able to trigger microglia activation, the resident immune cell of the CNS important in the pathogenesis of MS (Tanaka, 1989; Higashi et al., 2011).

#### Zn and MMPs

MMPs are a group of proteases all dependent of Zn for their proteolytic activity. They are involved in remodeling of the extracellular matrix and modifying cell–matrix interactions (Murphy and Nagase, 2008). Their proteolytic substrates include myelin basic protein, components of the blood–brain barrier (BBB), and neural/glial antigen 2 (NG2), important for proper remyelinisation in the CNS, giving them a potential important role in both the pathogenesis of MS as well as in the development of new treatments for MS (Chandler et al., 1995; Larsen et al., 2003; Ram et al., 2006). Studies measuring MMP levels in patients with MS have shown that several MMPs are altered (Bar-Or et al., 2003). A study by Kanesaka et al. (2006) showed that serum MMP-3 was significantly higher within 1 month before a relapse compared with the remission-phase serum levels of RRMS patients, suggesting a role in disease exacerbation through increased disruption of the BBB. The studies implicate an important role of especially MMP-9 in MS with results showing increased serum and cerebrospinal fluid (CSF) levels of MMP-9 in patients with MS compared with healthy controls (HCs) and increased MMP-9 CSF levels in RRMS patients during active disease compared with stable RRMS patients (Liuzzi et al., 2002). The involvement of MMP-9 in MS is further emphasized in a study by Correale and Bassani (2003) showing that MMP-9 levels in clinical isolated syndrome (CIS) patients that developed clinically definite MS was significantly higher than in those who did not.

The altered levels of MMPs may influence MS pathogenesis in several ways. First of MMPs may mediate migration of autoreactive lymphocytes across the BBB by their proteolytic degradation of the subendothelial basement membrane. This is shown by Leppers et al. (1995) in an *in vitro* model of the BBB with increasing levels of MMPs to result in increased migration of T-cells. Second, MMPs may contribute to myelin breakdown because of their proteolytic activity against myelin basic protein as well as contribute to epitope spreading of these. Even though MMP levels are highest during exacerbation of MS, it might also be important in the remission of the disease, as Larsen et al. (2003) showed by impairing remyelination in mice lacking MMP-9 because of their failure to clear NG2, a proteoglycan retarding the maturation and differentiation of oligodendrocytes. With all of the MMPs being Zn dependent for their proteolytic activity, local alterations in Zn level will most likely influence the effect of MMPs.

#### Zn altering therapy

With Zn's role in MS yet to be fully understood, it is still unclear whether altering Zn levels is helpful in inhibiting disease exacerbation. Studies of Zn supplementation have shown reduced T-cell activation and proliferation thereby being a promising approach in a future therapeutic manner (Stoye et al., 2012). Although a recent study showed no improvements of neurological signs in patients with MS compared with the placebo group after 12 weeks of Zn supplementation (Salari et al., 2015), another approach by Choi et al. (2013) with redistributing Zn in the body with Clioquinol (a Copper or Zn chelator) has shown promising results in the animal model of MS: Experimental Autoimmune Encephalitis (EAE). The study showed suppression of demyelination, reduced infiltration of encephalitogenic immune cells, inhibited BBB disruption, reduced MMP-9 activation and reduced clinical score of EAE after treatment with Clioquinol.

With the study by Wong et al. (1980) showing significantly reduced plasma Zn levels in patients with MS, studies have been performed since then studying Zn levels in patients with MS to unravel this potential role of Zn in the etiology or pathogenesis of MS.

As the amount of studies studying Zn levels in patients with MS accumulate and studies, with especially serum or plasma Zn levels showing heterogeneity in their results, a systematic review and a meta-analysis are performed to create an overview of the published results of Zn measuring studies in patients with MS.

## Methods

### Search Strategy and Inclusion Criteria

The literature-search was done in PubMed and EMBASE. The search profile for PubMed consisted of “Zinc AND ‘multiple sclerosis’” giving 133 hits and ‘Zinc AND ‘optic neuritis’” giving 14 hits with no overlap. The search profile for EMBASE consisted of “Zinc AND *multiple sclerosis” giving 141 hits and “Zinc AND *optic neuritis” giving 3 hits with no overlap. For both search profiles, all titles got examined and abstracts read of those maybe being relevant. Reference lists of retrieved studies were searched for additional reports. Inclusion criteria were studies having measured Zn levels in patients with MS.

### Meta-Analysis Inclusion Criteria

Inclusion criteria for studies in the meta-analysis were studies with results on serum or plasma Zn levels given in mean ± *SD* or *SEM*. With studies only giving results of subgroups, the weighted grand mean of the patients with MS included in the study was calculated, and the composite SD was found by analysis of variance.

### Meta-Analysis Statistical Method

Mean difference as well as 95% confidence intervals (CIs) were calculated using a random effects model instead of the fixed effect model, as heterogeneity between studies are expected. Random effect models assume and account for variable underlying effects in estimates of uncertainty, including both within-study and between-studies variance (Borenstein et al., 2009). The effect sizes were calculated with Review Manager (RevMan) [Computer program] Version 5.3., Copenhagen: The Nordic Cochrane Centre, The Cochrane Collaboration, 2014.

## Results

Of the 291 hits and of the reference lists in retrieved studies, 24 articles were found to study Zn levels in patients with MS, of which 19 had measured serum or plasma Zn levels and 6 had measured CSF Zn levels. A total of 11 studies were found to study other parameters of Zn or having data on subgroup analysis on Zn levels. The results of the 19 studies measuring serum or plasma Zn levels in patients with MS are summarized in Table 1. The results of the six studies measuring CSF Zn levels in patients with MS are summarized in Table 2, and the conclusions of the studies measuring other parameters or subgroup analyses on Zn levels are reported in Table 3. Results given in other units were converted to µmol/L with (Zn = 65.38 g/mol) (Wieser Michael et al., 2013) and results given with SEM were converted back to *SD*. It was found by Foley et al. (1968) that plasma Zn was 16% higher than in serum, although later studies have shown no significant or only very little difference between serum and plasma Zn concentrations, therefore, making them comparable (Kosman and Henkin, 1979; Kasperek et al., 1981; Kiilerich et al., 1981; Chen et al., 1986).

**Table 1.** Serum or Plasma Zinc in Multiple Sclerosis Versus Healthy Controls.

Study	Body fluid	MS (N)	HC (N)	Mean ± *SD* MS	Mean ± *SD* HC	*p*	Conclusion
Wong et al. (1980)	Plasma	26	39	n/a	n/a	<.0001	Significantly lowered in MS (M + F) if age < 50 years
Weismann et al. (1982)	Plasma	10	n/a	13 ± n/a	n/a	.01	Correlation between low plasma Zn and low albumin in MS
Palm and Hallmans (1982)	Serum	M: 21	M: 21	13.0 ± 1.9	14.8 ± 1.6	<.001	Significantly lowered in MS (M + F)
		F: 29	F: 29	12.1 ± 2.1	13.2 ± 1.6	.05	
Dore-Duffy et al. (1983)	Plasma	M: 18	M: 30	14.8 ± 3.2	12.5 ± 1.8	<.01	Only significantly higher in MS males
		F: 50	F: 30	12.3 ± 1.6	11.6 ± 1.5	NS	NS for MS (M + F)
		T: 68	T: 60	12.9 ± 2.4	12.0 ± 1.8	NS	
Ho et al. (1986)	Plasma	45	23	13.6 ± 3.1	13.5 ± 1.5	NS	NS
Kapaki et al. (1989)	Serum	15	28	15.8 ± 2.1	16.8 ± 2.9	NS	NS
Smith et al. (1989)	Plasma	14	33	15.7 ± 3.0	15.3 ± 0.1	NS	NS
Forte et al. (2005)	Plasma	60	60	91.7 ± 22.8	100.6 ± 15.5	≤.05	Significantly lowered in MS
Alimonti et al. (2007)	Serum	60	124	n/a	n/a	≤.0003	Significantly lowered in MS (M + F)
Masoud and Fakharian (2007)	Serum	M: 7	M: 7	12.0 ± 1.6	16.7 ± 1.6	<.0001	Significantly lowered in MS (M + F)
		F: 28	F: 28	13.3 ± 2.1	16.8 ± 1.1	<.0001	
		T: 35	T: 35	13.1 ± 2.1	16.8 ± 1.3	<.0001	
Gellein et al. (2008)	Plasma	9	16	5.1 ± 0.7	5.2 ± 0.9	NS	NS
Harbige et al. (2011)	Plasma	21	9	13.5 ± 2.5	13.1 ± 2.0	NS	NS
Ghazavi et al. (2012)	Serum	M: 17	M: 22	5.0 ± 3.3	20.2 ± 6.1	n/a	Significantly lowered in MS
		F: 43	F: 38	7.2 ± 5.4	20.2 ± 7.0	n/a	
		T: 60	T: 60	6.1 ± 4.9	19.5 ± 6.5	<.0001	
Al-Zubaidi (2012)	Serum	32	32	16.3 ± 3.3	23.0 ± 0.5	<.0001	Significantly lowered in MS
Nasrabadi et al. (2012)	Plasma	40	20	n/a	n/a	NS	NS
Sedighi et al. (2013)	Serum	58	39	36.7^a^ ± n/a	40.9^a^ ± n/a	.249	NS
Kazmierczak et al. (2014)	Plasma	11	38	n/a	n/a	.8	NS
Giacoppo et al. (2014)	Plasma	41	23	40.0 ± 9.6	43.0 ± 8.7	NS	NS
Ghoreishi et al. (2015)	Serum	50	50	1.1 ± 0.06	0.7 ± 0.07	<.005	Significantly higher in MS

*Note*. Mean and *SD* are given in µM. Different units have been converted to µM with (Zn = 65.38 g/mol), and SEM has been converted to *SD*. n/a = data not available; M = males; F = females; T = total; NS = non-significant; HC = healthy controls..

a Unit of measurement not available.

**Table 2.** Cerebrospinal Fluid Zinc in Multiple Sclerosis Versus Controls.

Study	Body fluid	MS (N)	C (N)	Mean ± *SD* MS	Mean ± *SD* HC	*p*	Conclusion
Linke et al. (1977)	CSF	40	24^a^	3.12 ± 2.20	1.12 ± 0.66	<.001	Significantly higher in MS.
Palm and Hallmans (1982)	CSF	M: 11	M: 11^b^	0.14 ± 0.03	0.17 ± 0.05	NS	NS
		F: 18	F: 18^b^	0.15 ± 0.06	0.16 ± 0.04	NS	
Ho et al. (1986)	CSF	MS active: 10	9^c^	0.55 ± 0.38	0.55 ± 0.45	NS	NS
		MS stable: 10		0.50 ± 0.38		NS	NS
Kapaki et al. (1989)	CSF	15	28^d^	0.53 ± 0.30	0.53 ± 0.08	NS	NS
Melo et al. (2003)	CSF	18	19^e^	0.29 ± 0.13	0.36 ± 0.21	NS	NS
Gellein et al. (2008)	CSF	9	16^f^	0.33 ± 0.09	0.38 ± 0.15	NS	NS

*Note*. Mean and *SD* are given in µM. Different units have been converted to µM (Zn = 65.38 g/mol), and SEM has been converted to *SD*. M = males; F = females; T = total; NS = non-significant; C = controls; Active = during attack.

a Patients in which cerebral disease has been excluded and with no deviation from standard levels.

b CSF samples from 18 HCs and the rest from patients with psychoneurosis, tension headache, and other pain syndromes in whom neurological examination failed to define an organic CNS lesion.

c Patients with other neurological diseases as Guillain-Barre disease, myasthenia gravis, central nervous system lupus erythematosus, bacterial meningitis, herpes encephalitis, amyotrophic lateral sclerosis, and pseudotumor cerebri.

d Patients cleared for neurological disease and with no sign of infection, liver disease, renal disease, tuberculosis, or alcoholism.

e Patients with no known neurological disorder or deficit.

f Patients suffering from myalgia, myelopathy, polyneuropathy, cervical stenosis, herpes zoster, and migraine.

**Table 3.** Other Parameters or Subgroup Analyses.

Study	Parameter	MS Total	HC Total	*p*	Conclusion
Wong et al. (1980)	Plasma Zn and clinical classification of MS	26	n/a	NS	No correlation between plasma Zn level and clinical classification of disease.
Palm and Hallmans (1982)	Serum Zn and disease stage (exacerbation, remission/steady state, slowly progressive) compared with HCs.	50	50	<.001	Serum Zn only significantly lowered in males with slowly progressive MS compared with HCs.
	Serum Zn and degree of malignancy of MS			<.001	Serum Zn only significantly lowered in males with a malignancy score between 1 and8 (>7 years duration, moderate/severe disability)
Rieder et al. (1983)	Whole blood Zn	119	35	<.01	Significantly higher whole blood Zn in MS compared with HCs.
	Whole blood Zn in male vs female MS cases			<.05	Significantly higher whole blood Zn in male MS cases compared with female MS cases.
	Whole blood Zn and disease MS subtype			.02	Significantly higher whole blood Zn in relapsing cases compared with chronic cases.
Dore-Duffy et al. (1983)	RBC bound Zn	68	60	<.05	RBC bound Zn significantly higher in MS compared with HCs.
	α2-macroglobulin bound Zn			<.01	α2-macroglobulin bound Zn significantly lowered in MS compared with HCs.
Ho et al. (1986)	Whole blood Zn	45	23	<.01	Whole blood Zn significantly higher in MS compared with HCs.
	RBC bound Zn			<.01	RBC bound Zn significantly higher in MS compared with HCs.
	Zn in ghost material of RBC membranes			<.01	Zn in RBC ghost significantly higher in MS and associated with the lipid-soluble fraction.
	RBC bound Zn and disease activity			<.01	RBC bound Zn significantly lower in exacerbating MS
Williams et al. (1988)	Plasma Zn and pressure sores	20		<.05	Significantly lower Zn in patients with MS who have pressure sores.
Smith et al. (1989)	RBC bound Zn	27	33	NS	No significant difference between MS and HCs, but RBC bound Zn tended to be higher in MS
	Steroid treatment and Zn levels			NS	No significant difference between steroid treated patients with MS and HCs, but serum and RBC Zn tended to be lower in MS.
Mauch et al. (1995)	Whole blood Zn	67	62	NS	Near identical whole blood Zn levels in MS and HCs.
Gellein et al. (2008)	Whole blood Zn	9	16	NS	No significant difference between MS and HCs, but whole blood Zn tended to be higher in MS.
Ghazavi et al. (2012)	Serum Zn and disease MS subtype	60		.009	Significantly lowered serum Zn in secondary progressive MS compared with RRMS.
Al-Zubaidi (2012)	Methylprednisolon treatment and serum Zn	96		<.0001	Significantly lowered serum Zn in treated patients with MS compared with non-treated patients with MS.
	Interferon-ß1 a treaments and serum Zn			<.0001	Significantly higher serum Zn in treated patients with MS compared with non-treated patients with MS.

*Note*. Degree of malignancy = Duration score × Disability level. Duration score: 1–3 years = 5, 4–6 years = 4, 7–9 years = 3, 10–15 years = 2, > 15 = 1. Disability level: mild = 2, moderate = 3, severe = 4. n/a = no data available, NS = non-significant, HC = healthy controls, RBC = red blood cells.

### Meta-Analysis

**Figure 1.**
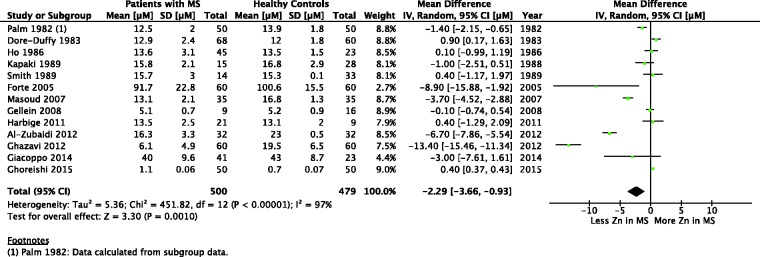
Forest Plot: Serum or Plasma Zinc in Multiple Sclerosis Versus Healthy Controls.

Of the 19 studies measuring serum or plasma Zn levels in patients with MS, 13 studies met inclusion criteria. The results of the meta-analysis is given in forest plot format in Figure 1 showing a significant difference in serum or plasma Zn levels in patients with MS versus HCs with a 95% CI of [−3.66, −0.93] and *p* = .001. An I^2^ value on 97% shows high heterogeneity in the meta-analysis.

## Discussion

The present meta-analysis of studies measuring serum or plasma Zn levels in MS reports a significant reduction in overall serum or plasma Zn levels in patients with MS, with studies reporting both lowered, no difference and increased serum or plasma Zn levels. Most of the studies included were in the non-significant range.

The great variance in the outcome of the studies might be explained by the heterogeneity of the serum or plasma Zn levels in patients with MS. The studies by Wong et al. (1980) and Palm and Hallmans (1982) show that the decrease in serum or plasma Zn with age in patients with MS does not correlate with the higher decrease seen in HCs with age. Studies including mostly old patients with MS may find no significant difference between MS and HCs even though it is possible that the younger patients with MS would show a significant difference. Therefore, it is not only enough for future studies to match patients with MS on age but also to be aware that a high mean age of patients with MS in the study might obscure a greater significant difference between younger patients with MS and HCs.

The study by Palm and Hallmans (1982) shows that the difference in correlation of decreasing serum or plasma Zn level with age between patients with MS and HCs is only found in males, as well as disease stage and degree of malignancy only having significant differences in Zn levels in male patients with MS. Therefore, some of the results showing no difference in serum or plasma Zn levels might be because of the low men or female MS patient ratio found in most of the studies. Because of the prevalence ratio of female versus men affected by MS being more than two to one, studies randomly selecting participants out of a MS population might find no significant results even though one is present in the male population of the participants. The results of Palm and Hallmans (1982) and Ghazavi et al. (2012) study not only emphasize the importance of keeping results between genders separated but also that the different subtypes of MS might have different alterations in serum or plasma Zn. Only the slowly progressive subgroup had significantly lowered Zn level in the study done by Palm and Hallmans and in the study done by Ghazavi et al., the patients with SPMS had significantly lowered Zn levels compared with the patients with RRMS. Future studies on serum or plasma Zn should, therefore, have in mind that uncritical mixing of patients with MS in studies on age, sex, disease subtype, duration of disease, and degree of disability might conceal a greater significant difference in subgroups of the study. It was examined whether it was possible to analyze the correlation between Zn levels and MS in regard to age or sex of patients and HC, although too few studies had their results presented in a way for this to be possible.

Not all studies included in the meta-analysis mentioned the proper precautions done to prevent the influence of the many confounders in serum or plasma Zn level analysis. As shown by Rea (1989), serum Zn levels are dependent on age and sex, though sex and age matching was not mentioned in 5 out of the 13 studies included (Kapaki et al., 1989; Smith et al., 1989; Gellein et al., 2008; Harbige et al., 2011; Giacoppo et al., 2014). As different medication here including Prednisolone, INF-ß1 a-treatment and oral contraceptives alter serum or plasma Zn levels, treatment with these should be paused before blood sampling in both patients and HCs or be an exclusion criterion (Briggs et al., 1971; Fallah et al., 2009; Al-Zubaidi, 2012). Precautions done against the possible confounding factor of medication in patients and HCs were only mentioned in 7 out of the 13 studies included (Palm and Hallmans, 1982; Dore-Duffy et al., 1983; Ho et al., 1986; Kapaki et al., 1989; Forte et al., 2005; Al-Zubaidi, 2012; Ghazavi et al., 2012).

The confounders mentioned so far are the most important in regard to the meta-analysis. The differences in the procedure of blood sampling and Zn analysis will not have a great impact on the result of the meta-analysis as long as the procedure has been the same between patients with MS and HCs and will mostly increase the heterogeneity between the studies. This is because of the use of the random effects model that also assumes and accounts for the regional differences in Zn levels (Iyengar, 1987). This is also the reason why the results of Forte et al. (2005), Gellein et al. (2008), Giacoppo et al. (2014), and Goreishi et al. (2015) were included, even though their results of plasma Zn are very different compared with the other studies. The low levels of serum or plasma Zn recorded in Gellein et al. and Goreishi et al. should result in severe Zn deficiency symptoms in both patients and HCs. Since the authors does not mention any reports of Zn deficiency as well as results in the four studies report Zn concentrations in the same levels for both patients and HCs, it is assumed that the low levels is a consequence of the analysis used in assessing the Zn concentration. It should be noted that the measuring of serum or plasma Zn levels includes many confounders here including circadian and postprandial fluctuations (Markowitz et al., 1985; Wallock et al., 1993) as well as being prone to contamination through hemolysis in blood samples and prolonged stasis while the blood sample is taken (Kiilerich et al., 1980). Information about precautions against these confounders are lacking in most studies.

As most of the studies included reported no significant difference in serum or plasma Zn levels in patients with MS, the risk of publication bias to have an influence on the outcome of the meta-analysis is assumed to be low.

Of the six studies measuring CSF Zn levels, the study by Linke et al. (1977) found a significant increase, though none of the later studies could find a significant difference between patients with MS and controls. It is hard to conclude anything on those studies since the high SD in most of the studies report big variance between the measurements and combined with the small number of participants it is highly unlikely to be able to find a statistical difference. The big SD and the variance in Zn levels between the studies may be explained be the small amount of Zn present in CSF fluid, making the measurements very susceptible to contamination during the analysis. It should be noted although that of the five studies done after Linke et al., the mean were in MS found either to be the same or slightly lower than in the control group.

Beside the significant decrease in plasma or serum Zn levels in patients with MS compared with HCs other interesting findings in regard to Zn's potential influence on MS pathogenesis so far is the alterations of whole blood Zn of patients with MS as reported by several studies as well as the increase in RBC Zn. Whole blood Zn was found to be higher in patients with MS, with male patients with MS having significantly higher levels compared with female patients and relapsing cases having increased levels as well compared with chronic cases as shown by Rieder et al. (1983). Some of the explanation for this increase is elaborated in the studies by Dore-Duffy et al. (1983) and Ho et al. (1986) showing increased amount of Zn in the lipid-soluble fraction of RBC membranes with results showing up to 3 to 4 times higher Zn in membranes of RBCs in patients with MS as well as an increased amount of Zn bound to α2-macroglobulin. With no difference in plasma Zn as well as cytoplasmic RBC Zn, they suggest that patients with MS have altered compartmentalization of Zn.

With the study by Ho et al. (1986) showing a dramatic reduction of these Zn levels during the exacerbation of an MS attack and slowly increasing Zn levels during the remission, together with the promising therapeutic effect on EAE by altering Zn levels with Clioquinol, alterations in local Zn levels may play an important role in the pathogenesis of MS. Future studies should examine whether the alteration in compartmentalization in Zn is only present in RBC or is also present in lymphocytes or other immune cells in patients with MS.

## Summary

A systematic review of Zn potential role in the pathogenesis of MS as well as a meta-analysis showing significantly lowered Zn levels in MS patients compared with healthy controls.
